# Prevalence, Molecular Identification, and Antimicrobial Resistance Traits of *Pasteurella multocida* Isolated From Nasal Swabs of Buffaloes in Bangladesh

**DOI:** 10.1002/mbo3.70432

**Published:** 2026-09-30

**Authors:** Piash Kumer Ghosh, Asikur Rahman, Milton Roy, Md Abdur Nur Sakib, Md Monjur‐e‐Alahi Tuha, Masud Ali, Md Shahidur Rahman Chowdhury, Md Bashir Uddin, Md Mahfujur Rahman, Md Mukter Hossain

**Affiliations:** ^1^ Department of Medicine, Faculty of Veterinary, Animal and Biomedical Sciences Sylhet Agricultural University Sylhet Bangladesh

**Keywords:** antimicrobial resistance, Bangladesh, buffaloes, molecular identification, multidrug resistance, nasal swabs, *Pasteurella multocida*, prevalence

## Abstract

*Pasteurella multocida* is an important bacterial respiratory pathogen of buffaloes and may cause major economic losses in the livestock sector. But there is a lack of data on its prevalence, risk factors, and antimicrobial resistance patterns in Bangladesh. The study was cross‐sectional, and a total of 400 buffaloes from seven locations in Bangladesh were included in the nasal swab collection. Isolation was done using culture methods and confirmed by polymerase chain reaction (PCR). Associations between infection and risk factors were explored using logistic regression analysis; antibiotic susceptibility was determined by the disc diffusion method. The co‐resistance patterns were assessed by Pearson's correlation analysis. The culture‐based prevalence of *P. multocida* was 12.25%, compared with a PCR‐confirmed prevalence of 7.25%; all PCR‐positive isolates were capsular type B, with notable regional variation in prevalence. Buffaloes with respiratory symptoms had significantly greater odds of being infected (OR = 8.88, *p* < 0.001), and the younger group of animals aged 1–3 years had a greater predisposition to infection than the younger ones, but sex was not significantly associated with infection. The isolates exhibited high antibiotic resistance, as demonstrated by antimicrobial susceptibility testing, particularly against ampicillin (96.55%), oxytetracycline (93.10%), and penicillin‐G (89.65%). Cefixime (93.10%) and azithromycin (68.96%) remained largely effective by contrast. Positive correlations were identified between resistance to β‐lactams, tetracyclines, and aminoglycosides. These findings suggest that multidrug‐resistant *P. multocida* type B is circulating in buffaloes in Bangladesh and highlight the need for increased surveillance, rational use of antimicrobials, and integrated control strategies.

## Graphical abstract

*Pasteurella multocida* was detected in buffalo habitats through culture (49 isolates) and polymerase chain reaction confirmation (29 isolates). Antibiotic susceptibility testing showed high ampicillin resistance, underscoring the emergence of antimicrobial resistance and emphasizing the need for improved surveillance and targeted therapeutic strategies.

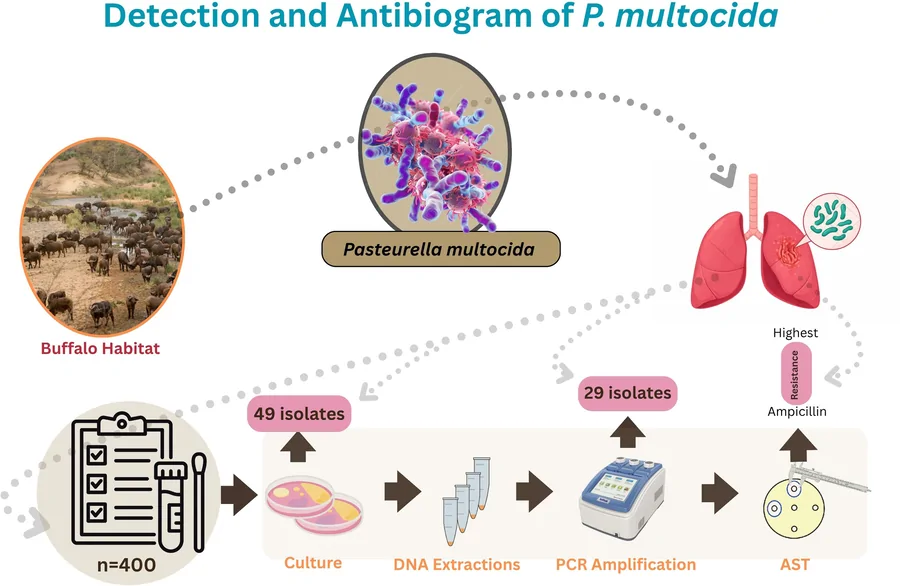

## 1 Introduction

The agricultural economy of Bangladesh is predominantly centered on agriculture and livestock raising, generating employment opportunities and ensuring adequate protein intake for the population (Ape et al. 2025). Buffaloes are crucial for livestock farming, significantly contributing to the economy and the livelihoods of rural populations by supplying milk, draught, and meat (Akbor et al. 2007). Buffalo trachea and lung tissues in Bangladesh exhibit significant respiratory lesions, highlighting the epidemiological importance of respiratory health and microbial colonization in this species (Akbor et al. 2007). Despite the economic significance of buffalo, managing their health remains difficult due to limited veterinary infrastructure, inadequate biosecurity measures, and emerging infectious diseases that affect both animal welfare and productivity (Ape et al. 2025; Pehan et al. 2025).

Nasal colonization is of epidemiological importance, as the upper respiratory tract serves as a crucial interface for microbial interactions between animals and their environment, enabling transmission through secretions, aerosols, and close contact within herds and farms (Lu et al. 2025; Ouyang et al. 2023). The nasal cavity serves as a significant reservoir for opportunistic infections, which may then disseminate to other regions of the respiratory system (Dimitri‐Pinheiro et al. 2020). It is well accepted that suppression of the host's immune system, caused by stress or infection, predisposes individuals to accelerated proliferation of opportunistic pathogens in the upper respiratory tract and diminishes clearance in the lower respiratory system, thereby initiating pathogenesis (Holman et al. 2015). Infections in the upper and lower respiratory tracts in buffalo, caused by bacterial agents, such as *Pasteurella multocida*, frequently impair development, reproduction, and increase mortality. *P. multocida* can proliferate in this region and disseminate to the lower respiratory tract, resulting in hemorrhagic septicemia (HS) or pneumonic pasteurellosis (Mohammed et al. 2018).

HS is a deadly disease that causes fever, edema, respiratory distress, and abrupt death in large ruminants. *P. multocida* serotypes B:2 and E:2 cause regular outbreaks in Asia, particularly Bangladesh, killing cattle and buffalo (Ara et al. 2016). The impact of husbandry techniques on the risk and dissemination of HS remains unknown. The majority of HS cases in cattle and buffaloes are acute or per‐acute and identified in the field. Owing to the persistence of organisms in the lymphatic tissues of the upper respiratory tract, both ill and recovered animals operate as carriers, intermittently shedding organisms in nasal secretions. Cattle and buffaloes are prevalent carriers of *P. multocida* that transmit the infection to other indigenous species (Shome et al. 2024). HS severity and speed may depend on age, sex, and environment. All ages and sexes are susceptible, although young growing males are most susceptible (Kabeta et al. 2015).

Antimicrobial resistance (AMR) is a significant worldwide health crisis in the 21st century, posing a threat to both human and veterinary medicine (Naghavi et al. 2024). Important factors that contribute to the development of AMR include the inappropriate and irrational use of antimicrobials in livestock, poultry, fish, and humans as well (Chowdhury et al. 2021). There are several modes of action by which antibiotics act in animals, which can affect their effectiveness and may even lead to the development of bacterial strains resistant to the antibiotic (Rasool et al. 2025). The misuse and overuse of antibiotics have led to the evolution of AMR and resistant bacteria and genes in all types of animal farming in Bangladesh due to inadequate veterinary healthcare, monitoring, and regulatory services, excessive informal animal health service providers, and the knowledge gaps of farmers on drugs (Al Amin et al. 2020). Recent research indicates that respiratory infections like *P. multocida* are becoming more resistant to conventional medications like trimethoprim‐sulfamethoxazole and macrolides, and carry several β‐lactamase and other resistance genes (Ali et al. 2025).

There is currently a lack of systematic information on the prevalence, molecular identification, and antibiotic susceptibilities of bacteria isolated from nasal swabs of buffaloes in Bangladesh. In Bangladesh, most research has focused on fecal carriage, mastitis, or mixed‐species investigations. The sample sizes for buffalo have been quite limited, and no extensive molecular characterization of nasal isolates has been available (Akter et al. 2018; Dashgupta et al. 2023; Gupta et al. 2018; Paul et al. 2010). To understand the reservoir of respiratory infections, guide clinical therapy, and design sensible AMR stewardship programs, it is vital to have more comprehensive data on nasal carriage in buffaloes. This work aimed to determine the prevalence of *P. multocida* isolated from buffalo nasal swabs in specific regions of Bangladesh, to validate their identity using molecular techniques, and to analyze their antibiotic susceptibility profiles. These findings are anticipated to offer core data for expanded diagnosis, treatment, and management of buffalo respiratory infections, as well as to aid national initiatives in monitoring AMR in food‐animal populations.

## 2 Materials and Methods

### 2.1 Ethical Statement

The study was conducted in accordance with the ethical standards of the Animal Experimentation and Ethics Committee (AEEC), Sylhet Agricultural University, Bangladesh (approval no. AUP2021052).

### 2.2 Study Area, Period, and Population

The study was conducted from September 2023 to August 2024 in six upazilas of Sylhet District (Zakiganj, Companiganj, Fenchuganj, Golapganj, Gowainghat, and Jaintapur) and Hatia Upazila (Swarna Dweep), Noakhali, Bangladesh (Figure 1). Buffaloes of different ages, sexes, and health conditions were included. A sample size of 385 was calculated assuming 50% prevalence, 95% confidence, and 5% precision (Faysal et al. 2025); consequently, 400 buffaloes were sampled.

**Figure 1:**
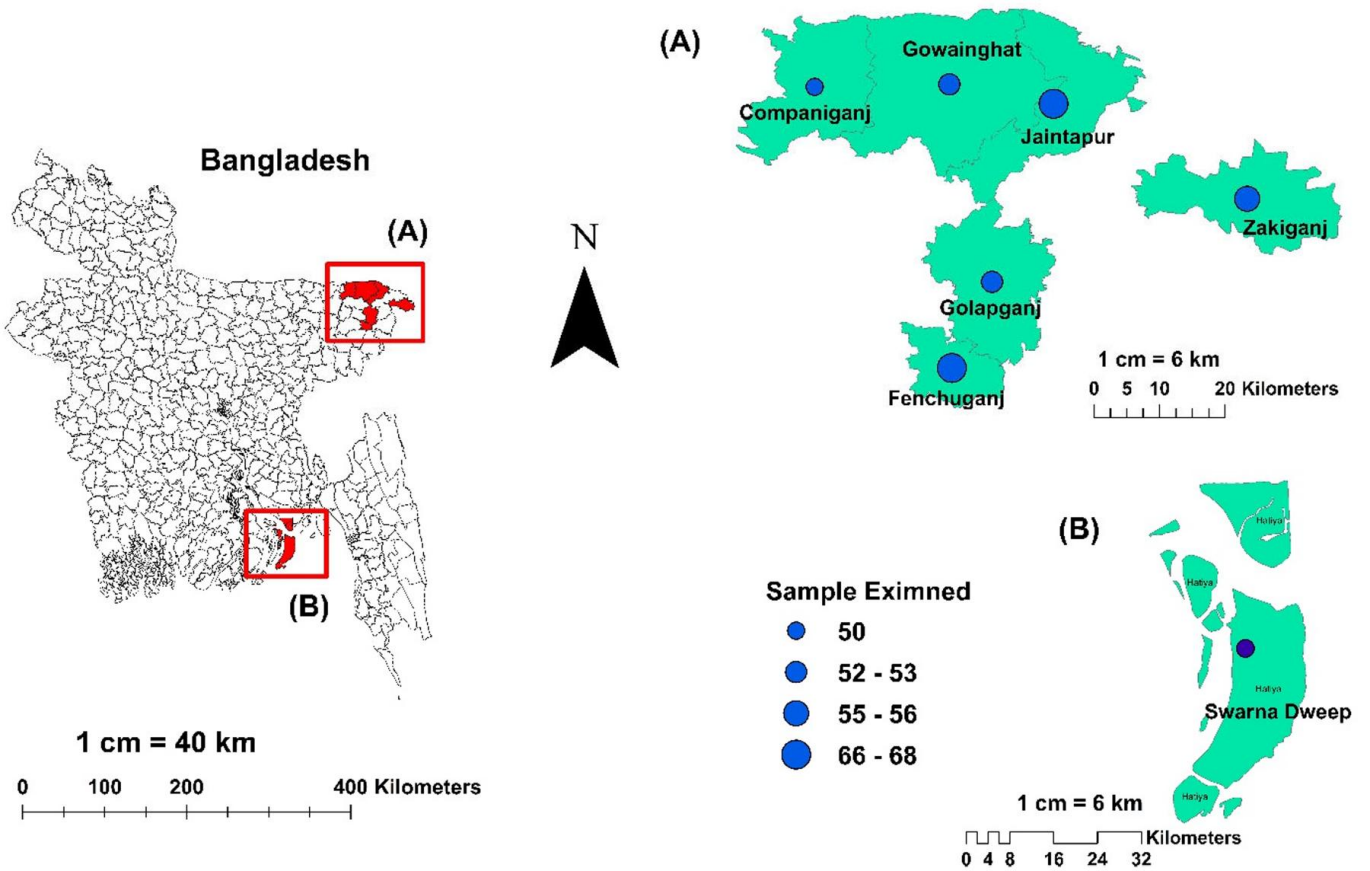
(A, B) The map showing the habitat of buffaloes and sampling size among study areas in Bangladesh was created using ArcMap 10.8.

### 2.3 Sample Collection and Transportation

Nasal swabs were collected aseptically from 400 buffaloes using sterile cotton swabs and placed in pre‐enrichment medium. Samples were labeled, transported in an icebox to the Department of Medicine, Sylhet Agricultural University, and maintained at 4°C until processing.

### 2.4 Isolation and Phenotypic Identification of *P. multocida*

Samples were enriched in selective brain heart infusion broth supplemented with 5% defibrinated sheep blood, amphotericin B, potassium tellurite, and gentamicin (GEN), and incubated at 37°C with 5%–10% CO_2_ for 16 h. Suspected colonies were identified based on colony morphology, Gram staining, and biochemical characteristics. Presumptive *P. multocida* isolates were stored in BHI broth containing 15% glycerol at −80°C until further analysis (Moore et al. 1994; Ali et al. 2025).

### 2.5 Molecular Confirmation and Capsular Typing

DNA was extracted using the Wizard Genomic DNA Purification Kit (Promega, USA). Polymerase chain reaction (PCR) was performed using *Pasteurella*‐specific primers for molecular confirmation, followed by capsular typing for types B and C using specific primer sets (Table 1). PCR conditions were based on Townsend et al. (2001), with the modifications described in the present study.

**Table 1:** Primer sequences used for the detection of *Pasteurella multocida* from buffaloes in this study.

Primers	Sequences (5′–3′)	Amplicon size (bp)	References
KMT1T7	F: ATCCGCTATTTACCCAGTGG	460	Shuka et al. (2022)
KMT1SP6	R: GCTGTAAACGAACTCGCCAC		
KTT72	F: AGGCTCGTTTGGATTATGAAG	620	Shuka et al. (2022)
KTSP61	R: ATCCGCTAACACACTCTC		
CAPE	F: TCCGCAGAAAATTATTGACTC	511	Shuka et al. (2022)
CAPE	R: GCTTGCTTGATTTTGTC		

### 2.6 Antimicrobial Susceptibility Testing of Obtained *P. multocida* Isolates

Antimicrobial susceptibility testing was performed using the Kirby–Bauer disc diffusion method on Mueller–Hinton agar. Bacterial suspensions were adjusted to a 0.5 McFarland standard and uniformly inoculated onto the agar surface. Antimicrobial discs (Hi‐Media Laboratories Pvt. Ltd., Mumbai, India) were used at the concentrations specified in Table S2. Following incubation at 37°C for 18–24 h, inhibition zones were measured in mm and interpreted according to the Clinical and Laboratory Standards Institute (CLSI 2022) guidelines. Multidrug‐resistant (MDR) isolates withstand three or more antibiotic classes (Rahman et al. 2024).

### 2.7 Agarose Gel Electrophoresis

PCR products were resolved by electrophoresis on 2% agarose gels prepared in 1× Tris‐Borate‐EDTA buffer at 100 V for 35 min. A 100‐bp DNA ladder was used to estimate amplicon size. Gels were stained with ethidium bromide (0.5 µg/mL) and visualized under ultraviolet illumination using an image documentation system.

### 2.8 Statistical Analysis

Data were entered and organized in Microsoft Excel 365 and analyzed using R version 4.4.2. Prevalence estimates and 95% confidence intervals were calculated using binomial methods (Tanni et al. 2025). Associations between categorical variables were assessed using the *χ* ^2^ test, followed by univariate logistic regression where appropriate (Asha et al. 2024). Results were presented as odds ratios (ORs) with 95% confidence intervals (CIs). Statistical significance was set at *p* < 0.05 (Sakib et al. 2025). Pearson's correlation coefficient was used to assess correlations among AMR patterns. Tables were generated using the gtsummary package (Sjoberg et al. 2022).

### 2.9 Geospatial Mapping and Visualizations

Study areas were visualized using ArcMap 10.8 (Esri, USA) with spatial data from DIVA‐GIS (www.diva-gis.org). R packages “ggplot2,” “circlize,” and “corrplot” were applied to create bar charts, circular heat maps, and a correlation matrix plot, respectively.

## 3 Results

### 3.1 Prevalence of *P. multocida*

Among 400 nasal swab samples collected from buffaloes, 49 were positive for *P. multocida* by bacteriological culture and biochemical screening, of which 29 were confirmed by PCR targeting the KTT72 and KTSP61 genes. The PCR‐confirmed prevalence was 7.25% (95% CI: 4.91–10.25), compared with 12.25% based on culture and biochemical screening (Figure 2).

**Figure 2:**
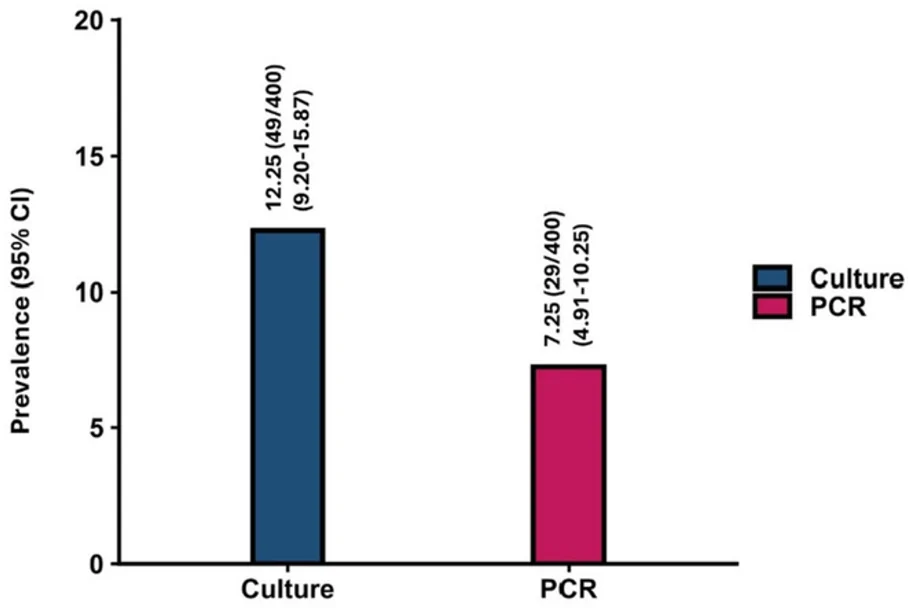
Overall prevalence of *Pasteurella multocida* in the respiratory tract of buffaloes. CI, confidence interval; PCR, polymerase chain reaction.

### 3.2 Geographical Distribution of *P. multocida*

By bacteriological culture, the prevalence varied across the study areas, ranging from 7.55% in Golapganj to 19.64% in Zakiganj. In contrast, no isolates (0.00%) were detected in Companiganj, whereas the highest 14.29% was reported in Zakiganj by molecular confirmation. No statistically significant association was observed among different study areas (Tables 2 and S2). Culture detected more positive samples than PCR in most research locations. Using capsular type‐specific PCR assays, molecular detection was performed, and the results showed that all PCR‐positive isolates were classified as *P. multocida* capsular type B infections.

**Table 2:** Prevalence and association of epidemiological variables with *Pasteurella multocida* in the respiratory tract of buffaloes.

Variable name	*n*/*N*	Prevalence (95% CI)	Logistic regression analysis			*χ* ^2^ value (df)	*p* value
			OR	95% Cl	*p* Value		
*Age*						5.27 (2)	0.072
< 1 year	3/112	2.68 (0.56–7.63)	*Ref*	—			
1–3 years	18/184	9.78 (5.90–15.02)	3.94	1.30–17.1	0.031		
> 3 years	8/104	7.69 (3.38–14.60)	3.03	0.85–14.1	0.11		
*Sex*						0.80 (1)	0.372
Female	13/218	5.96 (3.21–9.98)	*Ref*	—			
Male	16/182	8.79 (5.11–13.88)	1.52	0.71–3.30	0.3		
*Respiratory health status*						29.48 (1)	< 0.001
Healthy buffaloes	7/281	2.49 (1.01–5.07)	*Ref*	—			
Buffalo with respiratory symptoms	22/119	18.48 (11.96–26.64)	8.88	3.85–23.1	< 0.001		
*Study area*						NA	NA
Gowainghat	5/52	9.62 (3.20–21.03)	*Ref*	—			
Zakiganj	8/56	14.29 (6.38–26.22)	1.57	0.49–5.51	0.5		
Jaintapur	4/66	6.06 (1.68–14.80)	0.61	0.14–2.41	0.5		
Golapganj	1/53	1.89 (0.05–10.07)	0.18	0.01–1.17	0.12		
Fenchuganj	9/68	13.24 (6.23–23.64)	1.43	0.46–4.93	0.5		
Swarna Dweep	2/55	3.64 (0.44–12.53)	0.35	0.05–1.73	0.2		
Companiganj	0/50	0.00 (0.00–7.11)	NA	NA	NA		

Abbreviations: CI, confidence interval; *n*, positive sample; *N*, total sample; NA, not applied; OR, odds ratio; *Ref*, reference category.

### 3.3 Molecular Prevalence of *P. multocida* in Relation to Epidemiological Variables

The prevalence of *P. multocida* in buffaloes is influenced by several factors. Buffaloes aged between 1 and 3 exhibited the highest prevalence of *P. multocida* at 9.78% (95% CI: 5.90–15.02), with significantly higher odds of infection than those aged less than 1 year (OR = 3.94, 95% CI 1.30–17.1, *p* = 0.031). The prevalence of *P. multocida* varied from male buffaloes at 8.79 (5.11–13.88) to female buffaloes at 5.96 (3.21–9.98), and no significant association was found by *χ* ^2^ test and logistic regression analysis. Health status highlighted a significant association influencing the prevalence of *P. multocida* (*p* < 0.001). A similar pattern was observed in logistic regression analysis. Buffaloes with respiratory symptoms showed the highest prevalence of 18.48 (11.96–26.64), followed by healthy buffaloes at 2.49% (1.01–5.07). In addition, buffaloes with respiratory symptoms (OR = 8.88, 95% CI 3.85–23.1, *p* < 0.001) exhibited a significantly higher infection rate compared with healthy buffaloes (Table 2).

### 3.4 AMR Patterns of *P. multocida*

The overall results of the Kirby–Bauer disc diffusion method for antibiotic susceptibility testing are illustrated in Figure 3. *P. multocida* isolates exhibited the highest resistance to ampicillin (AMP) (96.55%), followed by oxytetracycline (OTC) (93.10%) and penicillin‐G (PCG) (89.65%). In contrast, higher resistance rates were also observed for streptomycin (79.31%), sulphamethoxazole–trimethoprim (65.52%), GEN (55.17%), and chloramphenicol (51.72%). Nonetheless, a moderate resistance rate was also reported in kanamycin (44.82%). Conversely, cefixime (CFM) (93.10%) demonstrated the highest susceptibility, while azithromycin was 68.96% (Figure 3 and Table S3).

**Figure 3:**
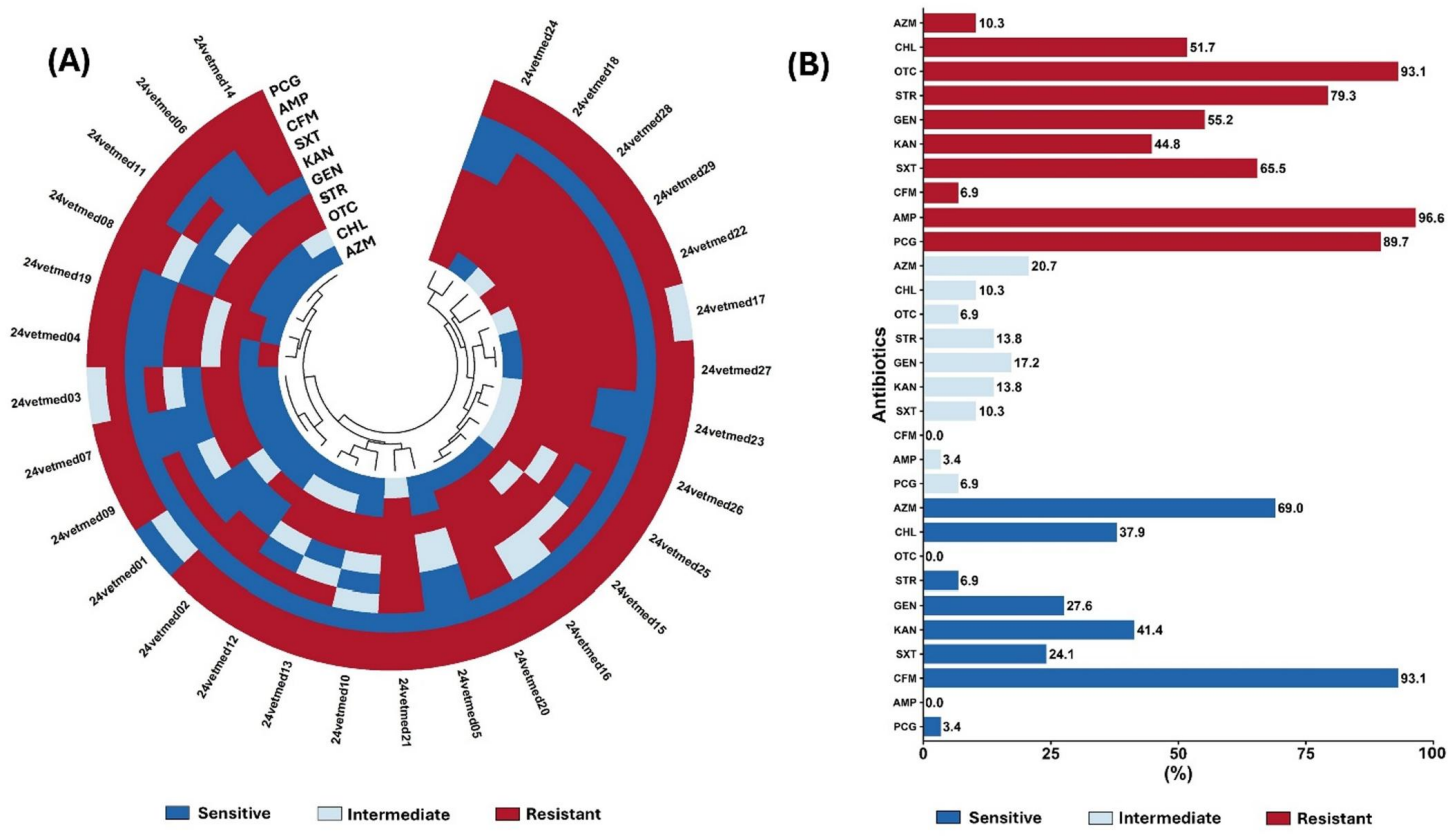
(A) Polar heat map shows the cluster analysis of the pattern of antibiotic susceptibility and (B) percentage distribution of sensitive, intermediate, and resistant responses against tested antibiotics of *Pasteurella multocida* isolated from the respiratory tract of buffaloes. AMP, ampicillin; AZM, azithromycin; CFM, cefixime; CHL, chloramphenicol; GEN, gentamicin; KAN, kanamycin; OTC, oxytetracycline; PCG, penicillin‐G; STR, streptomycin; SXT, sulphamethoxazole–trimethoprim.

### 3.5 Correlation Analysis in AMR Patterns

Pearson's correlation analysis revealed significant correlations in AMR patterns. A strong positive correlation between PCG and AMP (*r* = 0.81, *p* < 0.001) indicates high co‐resistance. GEN showed a robust connection with OTC (*r* = 0.80, *p* < 0.001). Additionally, AMP had a strong relationship with OTC (*r* = 0.69, *p* < 0.01) and a moderate one with streptomycin (*r* = 0.56). Moderate correlations were also found between GEN and kanamycin (*r* = 0.42) and azithromycin (*r* = 0.52). Negative correlations were rare, the most notable being between CFM and GEN (*r* = −0.40) (Figure 4). Overall, the findings indicate significant co‐resistance among antimicrobial classes, particularly β‐lactams and aminoglycosides, suggesting shared resistance mechanisms.

**Figure 4:**
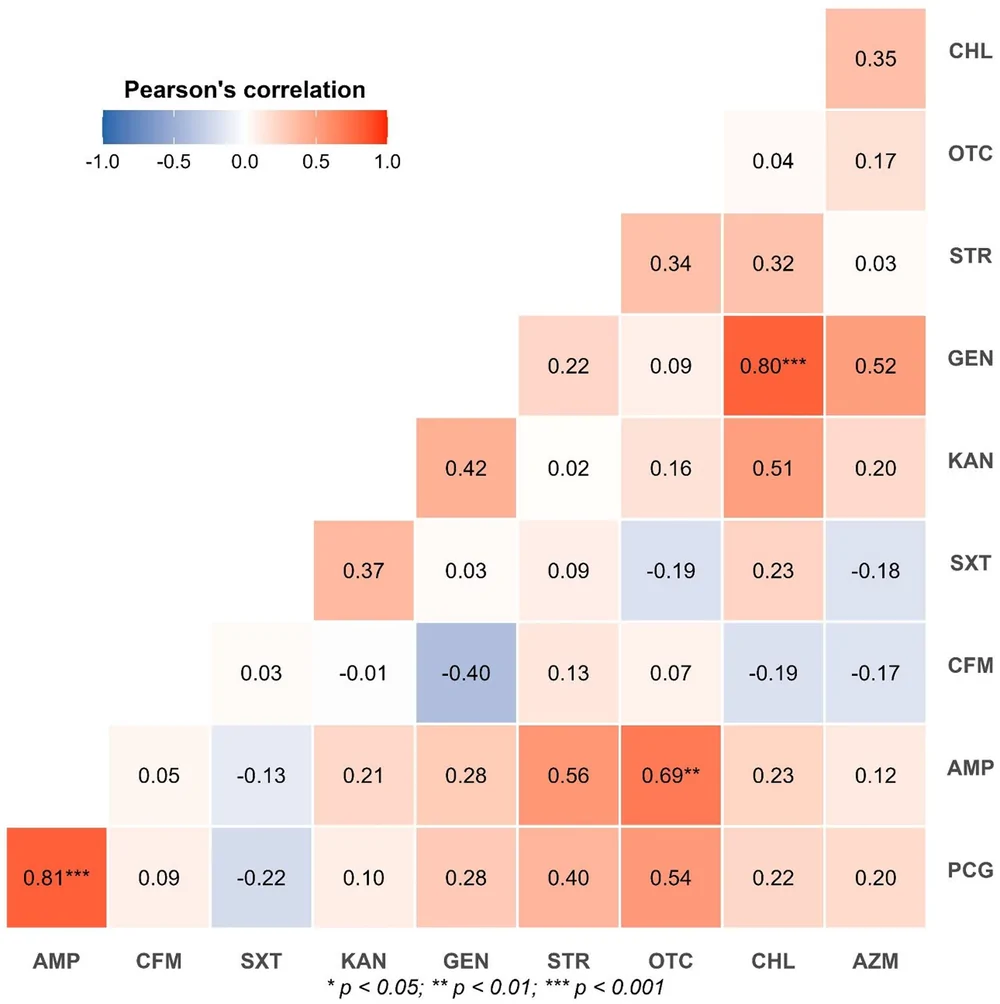
Pearson's correlation matrix among different tested antibiotics of *Pasteurella multocida* isolated from the respiratory tract of buffaloes. AMP, Ampicillin; AZM, Azithromycin; CFM, Cefixime; CHL, Chloramphenicol; GEN, Gentamicin; KAN, Kanamycin; OTC, Oxytetracycline; PCG, Penicillin‐G; STR, Streptomycin; SXT, Sulphamethoxazole–Trimethoprim.

## 4 Discussion

Pasteurellosis, a prevalent respiratory infection globally, is caused by the *Pasteurella* genus. *P. multocida* is the most commonly documented species within that genus (Hussein 2021). *P. multocida*, a prevalent bacterial pathogen in calves with respiratory disorders, is a commensal organism in the upper respiratory tracts of animals and has been associated with a rise in respiratory disease outbreaks in recent years (Abed et al. 2020; Ali et al. 2025; Calderón Bernal et al. 2022). This study aimed to determine the prevalence of *P. multocida* in buffalo nasal swabs from designated locations in Bangladesh, to confirm their identity using molecular techniques, and to evaluate their antibiotic susceptibility profiles.

This study indicates that the prevalence of *P. multocida* in buffaloes is 7.25% by PCR and 12.25% by culture. The lower proportion of PCR‐confirmed isolates compared with culture‐positive isolates may reflect differences in bacterial load, DNA quality, or the presence of PCR inhibitors, as well as the limitations of phenotypic identification. Therefore, the culture‐positive isolates should be considered presumptive, whereas PCR‐positive isolates represent molecularly confirmed *P. multocida*. Previous investigations have also reported discrepancies between culture and molecular detection, with PCR being more specific but sometimes less sensitive in field samples with low bacterial DNA proportion (OIE 2019). This outcome closely resembles the findings of Khamesipour et al. (2014) in Iran, who identified a *P. multocida* prevalence of 9%, which is close to the previously reported 3.08% in Egypt (Zaher et al. 2014). Our findings exceeded the prevalence rate previously published by Al‐Maary et al. (2017) in Saudi Arabia, which was 26.2%, and it is close to the prevalence rate reported by El‐Seedy et al. (2020) in Egypt, which was 18.2% in bovines. Ineffective immunization programs, delayed diagnosis, and insufficient antibiotic therapy may explain these results. However, El‐Jakee et al. (2016) and Elsayed et al. (2021) reported increases of 34.4% and 50%, respectively, in Egypt. Jamali et al. (2014) found 83.4% in North Iran.

Zakiganj (19.64% culture and 14.29% PCR) and Fenchuganj (17.65% culture and 13.24% PCR) showed the highest detection rates, despite significant variation in the distribution of infection across the research locations. Golapganj (7.55% culture and 1.89% PCR) and Jaintapur (7.58% culture and 6.06% PCR) exhibited rates that were lower than the global average. A culture positivity rate of 8.00% was observed in Companiganj, although there were no PCR‐positive samples (0.00%). Within the same framework, the disparities between the research regions might be accounted for by variations in management and hygiene settings.

Buffaloes with respiratory symptoms had roughly ninefold higher odds of infection than apparently healthy animals in this study. This supports the pathophysiology of *P. multocida*, a primary and opportunistic respiratory pathogen, and highlights the importance of respiratory illness as a clinical indication of infection. Buffaloes and cows in South and Southeast Asia that had difficulty breathing were more likely to test positive for *P. multocida* by culture or PCR (Shivachandra et al. 2011). A detrimental diet, poor transportation, changes in the weather, and the diseases that accompany them can weaken the body's defenses against *P. multocida*, which can then colonize and invade (Harper et al. 2015). Buffalo farms in resource‐limited nations usually lack good management or biosecurity; thus, these are very crucial.

Although not significant, age was weakly correlated with infection, suggesting that susceptibility may differ across age groups. Buffaloes aged 1–3 years showed a significantly higher infection probability compared with those under 1 year; however, buffaloes older than 3 years did not reveal significant differences. This means that young adult animals are susceptible to infection

Buffaloes aged 1–3 years may face increased risk due to immunological, physiological, and management problems. Animals in this age group often get stressed out by their habitat and care. This can happen when they stop nursing, move around more, interact with other animals, start working or preparing for work, or alter their habitat or diet. Stressors like these weaken the immune system and increase the likelihood of *P. multocida infection*, a respiratory disease that can occur at any time (Harper et al. 2006; Shivachandra et al. 2011).

In places where dams have been exposed or vaccinated, maternal antibodies may help protect calves under a year old. Passive immunity conferred by colostrum reduces the risk of pasteurellosis in young animals, but it wears off over time. This could be why younger individuals become sick less often. Buffaloes older than 3 years may not have a significant link, since they have been immune to pathogens. Adult animals that are endemic typically become immune, which could mean that infections take longer to develop or are less likely to occur at all. Quick changes in management may make elderly animals less likely to get hurt from stress.

The current study found no significant difference in infection rates between males and females, indicating that buffaloes were equally at risk. Males seemed more likely to be infected than females, but the difference was not statistically significant, and the OR confidence interval included 1. These results suggest that sex alone does not dictate infection risk. Past studies on pasteurellosis in buffaloes and cattle indicated that factors like age, health, stress, and management approaches had a bigger effect on the likelihood of infection than biological sex (Shivachandra et al. 2011). We found results similar to those of Bahr et al. (2020) and Gharibi et al. (2017). This may be because males are generally reared for meat production, which stresses them when switching from production to fattening. It could also be due to bad weather and lack of feed and drink while traveling. Buy animals from other markets and castrate them before they enter the feedlot. *P. multocida* may become more pathogenic after activation for any of these reasons (Bahr et al. 2020; Kabeta et al. 2015; Snowder et al. 2006).

Once pasteurellosis symptoms arise, treatment is ineffective and early diagnosis and treatment are crucial. Although *P. multocida* is usually susceptible to a wide variety of antibiotic classes, the prevalence of MDR *P. multocida* has been gradually increasing over the last several years (Shivachandra et al. 2011). The antimicrobial susceptibility testing of the 29 isolates showed that many were highly resistant to commonly used antibiotics, indicating that many of the spreading strains are resistant to several drugs. AMP, OTC, and PCG had the highest resistance rates. These are all common first‐line or empirical treatments in animal production systems. The high level of resistance to these drugs implies that they have been used for a long time and often without adequate care, which has likely exerted strong selective pressure on resistant strains (Petrocchi‐Rilo et al. 2020).

Antibiotic sensitivity tests on *P. multocida* isolates revealed resistance to AMP (96.55%), OTC (93.10%), and PCG (89.65%), which is supported by the reports (El‐Seedy et al. 2020; Michael et al. 2018), and it revealed that *P. multocida* isolates showed high resistance to AMP, penicillin, and OTC. Furthermore, our results align with those of Elsayed et al. (2021), who reported significant resistance to OTC (98.2%) and low‐level resistance to β‐lactam antibiotics (18.2%). (Cuevas et al. 2020) reported a significant resistance to penicillin (72.2%) and complete susceptibility to OTC infections (100%) in their investigation. The high resistance to PCG, AMP, and OTC in our study may have been due to negligent antibiotic administration without drug susceptibility testing or to virulent, resistant bacteria colonizing the respiratory system (Petrocchi‐Rilo et al. 2020). Overuse of tetracycline for *P. multocida* infection therapy and meta‐phylaxis has resulted in several tetracycline‐resistant strains (Babetsa et al. 2012).

On the other hand, most isolates were still very sensitive to CFM (93.10%) and moderately sensitive to azithromycin (68.96%). This shows that these antimicrobials are still more effective than others. The efficacy of third‐generation cephalosporins, such as CFM, may be attributed to their limited use in standard veterinary practice, thereby diminishing selective pressure. Macrolides, such as azithromycin, are also less commonly used in large ruminants, which could explain why resistance levels are lower. However, using these very vital antimicrobials too much can quickly lead to resistance.

Pearson's correlation analysis showed strong, biologically significant associations among AMR patterns, indicating that the isolates were likely to be resistant to multiple drugs simultaneously. PCG and AMP have a strong positive association, indicating that β‐lactam antibiotics are highly resistant to each other. This is most likely because they share resistance mechanisms, such as making β‐lactamase or changing the structure of penicillin‐binding proteins (Michael et al. 2018; Shivachandra et al. 2011).

The strong link between GEN and OTC, as well as the strong link between AMP and OTC, points to co‐selection of resistance features rather than direct mechanistic overlap. Tetracycline and aminoglycoside resistance genes are typically found together on mobile genetic elements, like plasmids or transposons. This allows them to be selected simultaneously under antimicrobial pressure (Michael et al. 2018; Schwarz et al. 2001). Negative correlations were not common, but the fact that CFM and GEN are negatively correlated implies that being resistant to aminoglycosides does not always make you less susceptible to third‐generation cephalosporins. This observation is consistent with prior reports: when cephalosporins are used less frequently in veterinary medicine, they remain effective against MDR *P. multocida* strains. Considering these data, it is clear that to effectively reduce pasteurellosis in buffalo populations, early diagnosis, targeted vaccination, improved management techniques, and prudent antibiotic use, accompanied by routine susceptibility testing, are all necessary. This study performed molecular characterization to confirm species and type the capsid only, but did not conduct more advanced genotypic investigations, such as virulence gene profiling, multi‐locus sequence typing, or whole‐genome sequencing. The study used phenotypic approaches to examine AMR but did not identify the genes or mobile genetic elements, which are limitations of this study.

## 5 Conclusion

This study offers a detailed understanding of the prevalence of *P. multocida* in buffalo populations in Bangladesh, as well as the risk factors and antibiotic resistance associated with the disease. Using culture and molecular identification, it is demonstrated that *P. multocida* is a significant respiratory pathogen. Geographical differences highlight how management techniques, sanitation practices, and local husbandry influence disease occurrence. The significance of early identification and treatment is underscored by the close link between respiratory symptoms and the infection. This reveals that young adult animals are a very high‐risk category, probably because they are under greater stress and face more management problems. Age and sex were not the most important factors, but buffaloes between the ages of 1 and 3 years were much more likely to be vulnerable. This shows that this group is at high risk. These results suggest that focused prevention interventions, such as vaccination and improved management during high‐risk periods, could greatly reduce the burden of disease. The fact that *P. multocida* isolates are often MDR is a major cause for concern. This is especially true for commonly used antibiotics, such as PCG, AMP, and OTC. Correlation research found many co‐resistance patterns, indicating that resistance determinants are most likely linked and selected together. This makes it very hard to give therapeutic interventions effectively. Even if CFM and azithromycin are still quite effective, it's crucial to use them carefully because they are important and could lead to resistance. All the data show an urgent need for greater antimicrobial stewardship, systematic antibiotic susceptibility testing before treatment, and stronger biosecurity and immunization initiatives. It will be necessary to integrate molecular diagnostics with AMR monitoring to control pasteurellosis and prevent the spread of resistant *P. multocida* strains in buffalo production systems.

## Author Contributions

**Piash Kumer Ghosh:** data curation, formal analysis, methodology, validation, writing – original draft, writing – review and editing. **Asikur Rahman:** data curation, validation, formal analysis, methodology, writing – original draft, writing – review and editing. **Milton Roy:** data curation, formal analysis, writing – original draft, writing – review and editing. **Md Abdur Nur Sakib:** formal analysis, methodology, validation, software, writing – original draft, writing – review and editing. **Md Monjur‐e‐Alahi Tuha:** data curation, formal analysis, methodology, validation, writing – original draft, writing – review and editing. **Masud Ali:** data curation, formal analysis, methodology, validation, writing – original draft, writing – review and editing. **Md Shahidur Rahman Chowdhury:** methodology, data curation, formal analysis, validation, writing – review and editing, writing – original draft. **Md Bashir Uddin:** conceptualization, formal analysis, investigation, supervision, writing – review and editing, writing – original draft. **Md Mahfujur Rahman:** conceptualization, formal analysis, validation, investigation, methodology, supervision, writing – original draft, writing – review and editing. **Md Mukter Hossain:** conceptualization, formal analysis, funding acquisition, investigation, methodology, validation, project administration, supervision, visualization, writing – review and editing, writing – original draft.

## Ethics Statement

The authors have nothing to report.

## Conflicts of Interest

The authors declare no conflicts of interest.

## Supporting information


**Table S1:** List of antibiotics used for antimicrobial susceptibility testing.
**Table S2:** Prevalence of *P. multocida* in buffalo respiratory tract according to study area.
**Table S3:** Antimicrobial resistance patterns of *P. multocida* isolated from respiratory tract of buffaloes.

## Data Availability

The data that support the findings of this study are available on request from the corresponding author. The data are not publicly available due to privacy or ethical restrictions.
