# Supplementary material for: Prevalence, Molecular Identification, and Antimicrobial Resistance Traits of Pasteurella multocida Isolated From Nasal Swabs of Buffaloes in Bangladesh
Source: Microbiologyopen. 2026 Sep 30;15(5):e70432. doi: 10.1002/mbo3.70432 (PMC13627257; doi:10.1002/mbo3.70432)
Supplement: Supplementary file 1 [file MBO3-15-e70432-s001.docx]

**Prevalence, Molecular Identification, and Antimicrobial Resistance Traits of *Pasteurella multocida* Isolated from Nasal Swabs in Buffaloes of Bangladesh**

**Supplementary Table 1:** List of antibiotics used for antimicrobial susceptibility testing.

| **Group Name** | **Antibiotics** |
| --- | --- |
| Penicillins | Penicillin-G (PCG, 10U), Ampicillin (AMP, 10 μg) |
| Cephalosporins | Cefixime (CFM, 10 μg) |
| Tetracyclines | Oxytetracycline (OTC, 30 μg) |
| Macrolide | Azithromycin (AZM, 30 μg) |
| Sulfonamides | Sulfamethoxazole-Trimethoprim (SXT, 1.25/23.75 μg) |
| Aminoglycosides | Gentamicin (GEN, 10 μg), Kanamycin (KAN, 30 μg), Streptomycin (STR, 25 μg) |
| Amphenicol | Chloramphenicol (CHL, 30 μg) |

**Supplementary Table 2:** Prevalence of *P. multocida* in buffalo respiratory tract according to study area

| Study area | N | n (%) [95% CI] | |
| --- | --- | --- | --- |
|  |  | Culture | PCR |
| Zakiganj | 56 | 11 (19.64) [10.23–32.43] | 8 (14.29) [6.38–26.22] |
| Gowainghat | 52 | 7 (13.46) [5.59–25.79] | 5 (9.62) [3.20–21.03] |
| Jaintapur | 66 | 5 (7.58) [2.51–16.80] | 4 (6.06) [1.68–14.80] |
| Golapganj | 53 | 4 (7.55) [2.09–18.21] | 1 (1.89) [0.05–10.07] |
| Fenchuganj | 68 | 12 (17.65) [9.47–28.80] | 9 (13.24) [6.23–23.64] |
| Swarna Dweep | 55 | 6 (10.91) [4.11–22.25] | 2 (3.64) [0.44–12.53] |
| Companiganj | 50 | 4 (8.00) [2.22–19.23] | 0 (0.00) [0.00–7.11] |
| Total | 400 | 49 (12.25) [9.20-15.87] | 29 (7.25) [4.91–10.25] |

N= Number of samples examined, n= Positive Sample, CI= Confidence Interval

**Supplementary Table 3:** Antimicrobial resistance patterns of *P. multocida* isolated from respiratory tract of buffaloes

| **Antimicrobial** | **Antimicrobial resistance** | | |
| --- | --- | --- | --- |
|  | **Sensitive (%)** | **Intermediate (%)** | **Resistant (%)** |
| Penicillin-G | 1/29 (3.44) | 2/29 (6.89) | 26/29 (89.65) |
| Ampicillin | 0/29 (0.00) | 1/29 (3.44) | 28/29 (96.55) |
| Cefixime | 27/29 (93.10) | 0/29 (0.00) | 2/29 (6.89) |
| Sulphamethoxazole-Trimethoprim | 7/29 (24.13) | 3/29 (10.34) | 19/29 (65.52) |
| Kanamycin | 12/29 (41.37) | 4/29 (13.79) | 13/29 (44.82) |
| Gentamicin | 8/29 (27.58) | 5/29 (17.24) | 16/29 (55.17) |
| Streptomycin | 2/29 (6.89) | 4/29 (13.79) | 23/29 (79.31) |
| Oxytetracycline | 0/29 (0.00) | 2/29 (6.89) | 27/29 (93.10) |
| Chloramphenicol | 11/29 (37.93) | 3/29 (10.34) | 15/29 (51.72) |
| Azithromycin | 20/29 (68.96) | 6/29 (20.68) | 3/29 (10.34) |
